# Expression of Plasmid-Based shRNA against the E1 and nsP1 Genes Effectively Silenced Chikungunya Virus Replication

**DOI:** 10.1371/journal.pone.0046396

**Published:** 2012-10-08

**Authors:** Shirley Lam, Karen Caiyun Chen, Mary Mah-Lee Ng, Justin Jang Hann Chu

**Affiliations:** 1 Laboratory of Molecular RNA Virology and Antiviral Strategies, Yong Loo Lin School of Medicine, National University Health System, National University of Singapore, Singapore; 2 Flavivirology Laboratory, Department of Microbiology, Yong Loo Lin School of Medicine, National University Health System, National University of Singapore, Singapore; George Mason University, United States of America

## Abstract

**Background:**

Chikungunya virus (CHIKV) is a re-emerging alphavirus that causes chikungunya fever and persistent arthralgia in humans. Currently, there is no effective vaccine or antiviral against CHIKV infection. Therefore, this study evaluates whether RNA interference which targets at viral genomic level may be a novel antiviral strategy to inhibit the medically important CHIKV infection.

**Methods:**

Plasmid-based small hairpin RNA (shRNA) was investigated for its efficacy in inhibiting CHIKV replication. Three shRNAs designed against CHIKV Capsid, E1 and nsP1 genes were transfected to establish stable shRNA-expressing cell clones. Following infection of stable shRNA cells clones with CHIKV at M.O.I. 1, viral plaque assay, Western blotting and transmission electron microscopy were performed. The *in vivo* efficacy of shRNA against CHIKV replication was also evaluated in a suckling murine model of CHIKV infection.

**Results:**

Cell clones expressing shRNAs against CHIKV E1 and nsP1 genes displayed significant inhibition of infectious CHIKV production, while shRNA Capsid demonstrated a modest inhibitory effect as compared to scrambled shRNA cell clones and non-transfected cell controls. Western blot analysis of CHIKV E2 protein expression and transmission electron microscopy of shRNA E1 and nsP1 cell clones collectively demonstrated similar inhibitory trends against CHIKV replication. shRNA E1 showed non cell-type specific anti-CHIKV effects and broad-spectrum silencing against different geographical strains of CHIKV. Furthermore, shRNA E1 clones did not exert any inhibition against Dengue virus and Sindbis virus replication, thus indicating the high specificity of shRNA against CHIKV replication. Moreover, no shRNA-resistant CHIKV mutant was generated after 50 passages of CHIKV in the stable cell clones. More importantly, strong and sustained anti-CHIKV protection was conferred in suckling mice pre-treated with shRNA E1.

**Conclusion:**

Taken together, these data suggest the promising efficacy of anti-CHIKV shRNAs, in particular, plasmid-shRNA E1, as a novel antiviral strategy against CHIKV infection.

## Introduction

Chikungunya virus (CHIKV) is an alphavirus member from the family *Togaviridae*. CHIKV causes acute infection in humans with clinical symptoms characterized by sudden-onset chills and fever, headache, maculopapular rash and persistent arthralgia [1], [2]. The virus was first isolated from the serum of a febrile patient from Tanzania in 1952 [3]. In the 1960s, CHIKV was mainly transmitted to human populations in Africa and many parts of Southeast Asia by *Aedes aegypti* mosquitoes [2], [4]. In recent years from 2004–2007, major outbreaks of CHIKV infection in Kenya, India and islands in Indian Ocean have involved a second vector, *Aedes albopictus* [5], [6]. Since then, the virus has spread to areas predominated with *Aedes albopictus*, including Indonesia [1], Malaysia [7] and Singapore [8]. The rapid re-emergence of this viral pathogen has raised several concerns on its adaptability to new mosquito vectors and the risk of a CHIKV world pandemic [1]. In view of this, antiviral controls and strategies against CHIKV replication are urgently required. However, there is no specific treatment or clinically approved vaccine to date [8], [9]. Current therapies for CHIKV infection are symptomatic, including chloroquine, corticosteroids and non-steroidal anti-inflammatory drugs [9], [10]. Early CHIKV vaccine development and clinical trials have shown varying success [11]–[13], while other promising studies in human and animals using live attenuated CHIKV vaccine candidates are still currently in the midst of preclinical development and approvals [14], [15].

In view of the lack of effective pharmacological treatment for CHIKV disease, a potential antiviral strategy using antisense-mediated gene silencing of CHIKV replication may be investigated. CHIKV is a small (60–70 nm in diameter), spherical, enveloped virus with a single-stranded, positive-sense RNA genome of approximately 11.8 Kb [4]. The RNA genome encodes four non-structural proteins (nsP1–4), three structural proteins (Capsid, E1, E2) and two small cleavage products (E3 and 6K) [4]. In brief, nsP1–4 proteins are important for the synthesis of the viral RNA while Capsid, E1 and E2 proteins are required to form the mature CHIKV virion and are crucial in virus uncoating and assembly in infected host cells. As virus replication is heavily dependent on the host’s protein expression machinery, small interfering RNA (siRNA) known to induce specific viral mRNA knockdown have the potential to exert an inhibitory effect [16]–[18]. siRNA molecules of 21–23 nucleotides mediate the process of RNA interference (RNAi), an innate gene regulatory mechanism highly conserved in eukaryotes [19]. When the RNAi mechanism is induced by exogenous siRNAs, this results in the assembly of RNA-induced silencing complex (RISC) which degrades specific complementary mRNA molecules and thereby inhibits subsequent protein expression.

In the present study, we investigated the efficacy of plasmid-based small hairpin RNA (shRNA) against CHIKV replication in three CHIKV-permissive cell lines, namely, HeLa, RD and BHK cells. Expression of shRNAs from the shRNA-plasmid construct results in their intracellular processing to siRNAs, which in turn cause specific knockdown of viral RNA and inhibit subsequent viral protein expression (Figure S1). Based on the complete genome of Indonesia 0706aTw CHIKV strain, three anti-CHIKV shRNAs, namely, shRNA Capsid, E1 and nsP1 and their respective scrambled shRNA controls were designed (Table 1). The shRNA target sequences were determined by BLAST identification of sequence with complete homology to several different geographical CHIKV strains. Following CHIKV infection, stable shRNA E1 and nsP1-expressing cell clones demonstrated strong efficacy against CHIKV replication while shRNA Capsid cell clones displayed only a modest anti-CHIKV inhibitory effect. shRNA E1 was found to reduce CHIKV titre in HeLa, RD and BHK cell clones, thus suggesting that shRNA-mediated inhibition of CHIKV replication was not likely to be cell-type specific. Upon CHIKV infection of stable HeLa cell clones, shRNA E1 showed similar inhibitory trends at multiplicity of infection (M.O.I.) of both 1 and 10. In addition, shRNA E1 was found to exert strong anti-CHIKV specificity and CHIKV resistant-mutant strains were not generated after extensive viral passage in stable HeLa cell clones. Notably, pre-treatment of mice with shRNA E1 conferred a sustained protective effect from CHIKV infection.

**Table 1:** Respective shRNA target sequences in CHIKV Capsid, E1 and nsP1 genes.

shRNA designed^a^	Target sequence^b^ (21-mer)	Sequence location^c^
Capsid (Cap)	AAGAATCGGAAGAATAAGAAG	7743–7761
Scrambled Capsid(sCap)	AAGATAGCGGAAATAGAAAGA	–
E1	AAAGACGTCTATGCTAATACA	10614–10632
Scrambled E1 (sE1)	AAGAATCGCATGCTATATCAA	–
nsP1	AAGGCTAAGAACATAGGATTA	694–712
Scrambled nsP1(snsP1)	AAGCGGAAATACATGAGTATA	–

shRNAs were designed against CHIKV structural genes (Capsid and E1) and non-structural gene (nsP1) and were named accordingly to its gene target^a^. According to the guidelines for designing an effective shRNA construct, the antisense target sequence was incorporated with an additional AA dinucleotide at its 5′ end and has an optimal length of 21-mer. In the scrambled shRNA controls, underlined nucleotides refer to mismatch to its original target sequence^b^. Sequence location refers to the location of the shRNA target sequence in the Indonesia 0706aTw CHIKV genome^c^.

## Materials and Methods

### Design of Plasmid-based shRNA Construct against CHIKV Genes

Anti-CHIKV shRNAs were designed based on the CHIKV isolate, 0706aTw from Indonesia (GenBank; FJ807897). Potential target sites in CHIKV Capsid, E1 and nsP1 genes were located using siRNA Target Finder software (Ambion, USA) and three optimal gene sequences were further selected based on the manufacturer’s protocol and guidelines described by Ui-Tei and co-workers [20] (Table 1). To design highly effective plasmid-based shRNA constructs, the shRNA target sequences were also checked for 100% homology against the complete genomic sequences of different geographical CHIKV strains, namely, the Indonesia 0706aTw (GenBank; FJ807897), Singapore LK(EH)CH6708 (GenBank; FJ513654), Singapore 0611aTw (GenBank; FJ807896), India RGCB355/KL08 (GenBank; GQ428214) and Sri Lanka LK(EH)CH20108 (GenBank; FJ513679) of the East/Central/South African (ECSA) genotypes, S27-African prototype (GenBank; AF369024) of the Central/East African (CEA) genotype and Malaysia MY002IMR/06/BP of the Asian genotype using the nucleotide BLAST tool provided by NCBI (http://blast.ncbi.nlm.nih.gov/Blast.cgi?CMD=Web&PAGE_TYPE=BlastHome). Scrambled sequences of the anti-CHIKV shRNAs were designed as experimental controls and they were named as scrambled shRNA Capsid (sCapsid), scrambled shRNA E1 (sE1) and scrambled shRNA nsP1 (snsP1), respectively. Each shRNA construct is a 55-mer stem-and-loop duplex made up of a sense (19-mer) and antisense (21-mer) shRNA template DNA sequence separated by an intervening loop TTCAAGAGA (9-mer). The construct was cloned into pSilencer 4.1-CMV neomycin vector via BamH I and Hind III restriction sites at the 5′ and 3′ end, respectively. Following DNA amplication in cultured bacterial cells, shRNA-plasmid construct was extracted and purified by High-Speed Plasmid MINI kit (Geneaid, Taiwan).

The ligation of shRNA construct into the DNA plasmid vector was verified by DNA sequencing. Using ClustalW2 alignment, the sequence and orientation of the shRNA insertion in the plasmid was confirmed to ensure the proper expression and correct folding of shRNA in the stable cell clones (Figure S2 and Figure S3). The flanking sequences of shRNA inserts (ie. CMV promoter sequence and SV40 polyA signal sequence) and the loop sequence within the insert were also checked to ensure that their integrity were maintained after molecular cloning procedures (Figure S3). Scrambled shRNA Capsid, E1 and nsP1 inserts in the pSilencer construct were also verified (data not shown).

**Table 2:** Homology alignment of shRNA E1 target sequence to the CHIKV genome of several geographical isolates.

CHIKV isolate	Homologous sequence alignment	Location^a^
Indonesia0706aTw	Query AAAGACGTCTATGCTAATACASbjct GAAGACGTCTATGCTAATACA	10614–10632
Singapore 0611aTw	Query AAAGACGTCTATGCTAATACASbjct AAAGACGTCTATGCTAATACA	10624–10644
SingaporeLK(EH)CH6708	Query AA**A**GACGTCTATGCTAATACASbjct AA**C**GACGTCTATGCTAATACA	10601–10621
India RGCB356/KL08	Query AAAGACGTCTATGCTAATACASbjct AAAGACGTCTATGCTAATACA	10605–10625
Sri Lanka LK(EH)CH20108	Query AAAGACGTCTATGCTAATACASbjct AAAGACGTCTATGCTAATACA	10599–10619
S27-Africanprototype	Query AAAGACGTCTATGCTAA**T**ACASbjct AAAGACGTCTATGCTAA**C**ACA	10624–10644
Malaysia MY002IMR/06/BP	Query AA**A**GACGTCTATGCTAATACASbjct GG**C**GACGTCTATGCTAATACA	10601–10621

Nucleotide BLAST sequence analysis of shRNA E1 target site sequence (Query) to the genomic sequence (Sbjct) of the various geographical strains of CHIKV revealed 100% homology of the target site to the respective E1 gene sequence (except one base pair mismatch to the S27-African prototype, Singapore LK(EH)CH6708 and Malaysia MY002IMR/06/BP strains. The mismatched base is bold and underlined). An additional AA dinucleotide was incorporated to the 5′ end of the 19-mer shRNA to obtain an effective 21-mer shRNA construct. Alignment data for shRNA Capsid and shRNA nsP1 are not shown.

a Nucleotide position of the Sbjct sequence in the CHIKV genome.

**Figure 1:**
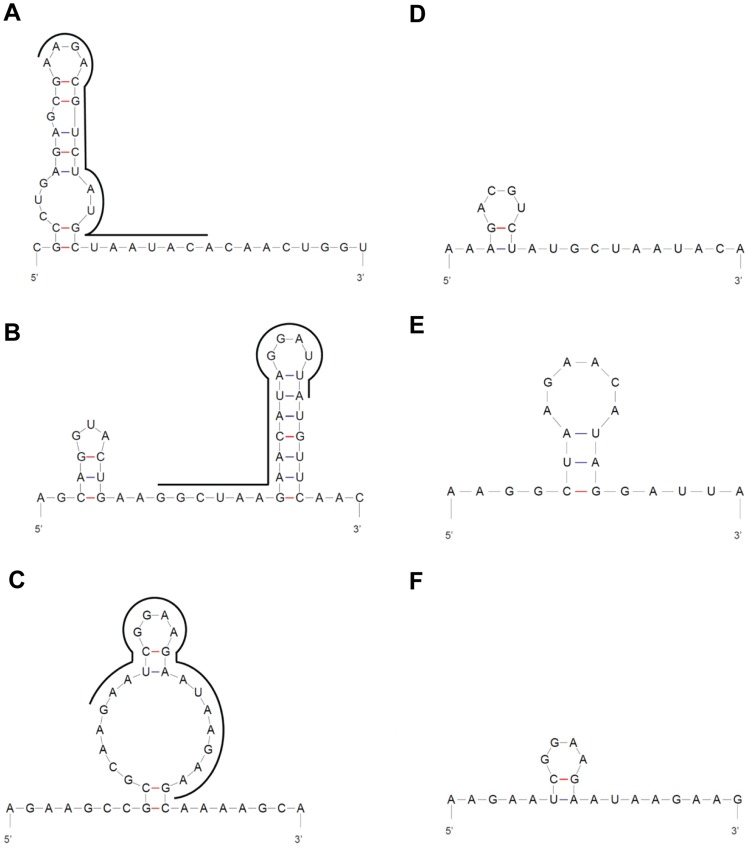
Predicted structures of shRNA target sequences in CHIKV genomic RNA and anti-CHIKV shRNA sequences designed. (**A**) Region of shRNA E1 target site (location: 10614–10632), (**B**) Region of shRNA nsP1 target site (location: 694–712) and (**C**) Region of Capsid shRNA target site (location: 7743–7761). The exact target site in each region is indicated by black bold lines. (**D–F**) shRNA was expressed from the pSilencer vector as an oligonucleotide duplex construct (55-mer) that contains the antisense sequence which is complementary to its target in the CHIKV genome. Antisense sequences of (**D**) shRNA E1, (**E**) shRNA nsP1 and (**F**) shRNA Capsid shown here are not predicted to form considerable secondary structures. All structures are predicted using mfold web server [29].

### Cell Culture and Transfection of Plasmid-shRNA Construct

In this study, HeLa cells (ATCC No. S3), Baby Hamster Kidney BHK-21 cells (ATCC No. CCL-10) and Human muscle rhabdomyosarcoma RD cells (ATCC No. CCL-136) were obtained from American Type Culture Collection. HeLa cells and RD cells were respectively cultured in DMEM medium and BHK cells were cultured in RPMI-1640 medium. All cell culture media were supplemented with 10% FCS and cells were incubated at 37°C in 5% CO_2_. One day prior to transfection of plasmid-shRNA constructs, HeLa, RD or BHK cells were seeded at 90% confluency on 6-well tissue culture plate. Following the manufacturer’s recommendation, 5 ng/ul of plasmid-shRNA was added with Lipofectamine LTX Reagent (Invitrogen, California, USA) to each well. In the next 3–4 weeks, positive shRNA-expressing cells were selected using G418 antibiotic (PAA Laboratories, Austria) at a previously optimized concentration of 800 µg/ml and stable shRNA cell clones of HeLa, RD and BHK were established.

### CHIKV Infection Assay and CHIKV Growth Kinetics

CHIKV virus used in this study was isolated from the serum of a CHIKV-infected patient (Singapore/07/2008 strain; Genbank; FJ513654) kindly provided by A/P Raymond Lin from National Public Health Laboratory, Ministry of Health, Singapore. CHIKV Ross strain of the ECSA genotype (Genbank; AF490259) used was kindly provided by A/P Ooi Eng Eong from Duke-NUS, Singapore. 24 h prior to infection, shRNA stable cell clones and the non-transfected HeLa, RD or BHK cells were seeded at 80–90% confluent monolayer on 24-well tissue culture plates. CHIKV infection at M.O.I. of 1 or 10 was performed with incubation of the appropriate amount of virus supernatant onto cell monolayer at 37°C in 5% CO_2_ for 1.5 h, with gentle rocking at every 15 min. Residual unbound viruses were washed off twice with PBS followed by incubation of the cells in DMEM media with 2% FCS at 37°C in 5% CO_2_. Supernatants were harvested at Day 1, 2 and 3 post-infection (p.i.) for quantification via viral plaque assays.

**Figure 2:**
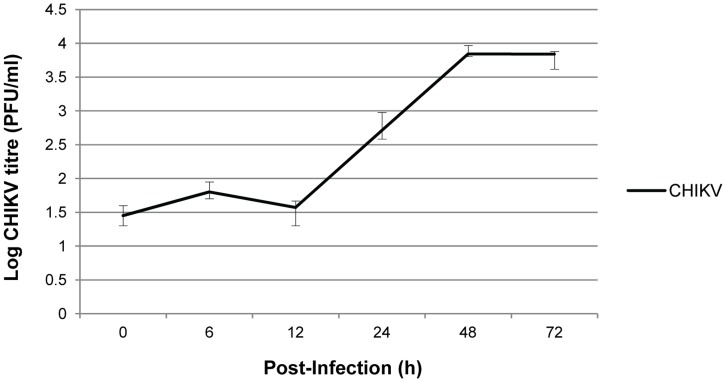
Growth kinetics of CHIKV in HeLa S3 cell line. CHIKV infection was performed at M.O.I. 1 and viral supernatants of the infected cells were harvested at the respective time points. Viral plaque assays were performed to quantitate the infectious CHIKV titre produced. The average ±S.E. (standard error) is expressed from three independent set of experiments.

To investigate the susceptibility of HeLa cells against CHIKV replication, CHIKV growth kinetics (at M.O.I. 1) was established. The procedure was similar to the above-mentioned CHIKV infection assay. Following CHIKV infection, cells were examined peridiocally under light microscopy for the presence of CHIKV-induced cytopathic effect (CPE) and supernatants were harvested at fixed time points of 0 h, 6 h, 12 h, 24 h, 48 h and 72 h for the quantification of infectious CHIKV titre via plaque assays.

### Viral Plaque Assay

On 24-well plate with 90% BHK cells monolayer, CHIKV infection was performed with 10-fold serially diluted supernatants. Cells were then incubated at 37°C in 5% CO_2_ for 1.5 h, with gentle rocking at every 15 min. Following washing with PBS twice, overlay media (1% Carboxymethyl cellulose and 1xRPMI-1640 supplemented with 2% FCS) was added to the cells. At Day 3 p.i., cells were stained with 10% paraformaldehyde/1% crystal violet solution. Virus titre in PFU/ml was quantitated by the number of plaques formed per well.

### Western Blot Analysis

Cell lysates of CHIKV-infected stable HeLa cell clones and non-transfected HeLa cell controls were harvested at Day 1, 2 and 3 p.i. and subjected to SDS-PAGE in 10% polyacrylamide gel. Bands were probed for CHIKV E2 protein expression using rabbit polyclonal anti-CHIKV E2 (CH13893 B3 generated in our laboratory) and secondary goat anti-rabbit alkaline phosphatase antibody (Millipore, USA). β-actin detection was performed using rabbit polyclonal anti-actin and mouse monoclonal anti-actin (Millipore, USA). The blot was developed by colorimetric method.

### Transmission Electron Microscopy

Stable shRNA E1 and nsP1 cell clones, scrambled cell clones and non-transfected HeLa cells seeded at 90% confluency in T75 cm^2^ tissue culture flasks followed by CHIKV infection. At Day 3 p.i., cells were fixed with 2.5% glutaraldehyde (Agar Scientific, Stansted, UK) at 4°C for 20 min, before being scraped and fixed at 4°C overnight. The fixed cells were then centrifuged and the pellet was washed with PBS and deionised water. The cell pellet was post-fixed with 1% osmium tetroxide (Ted Pella, Redding, California, USA) and 1% potassium ferro-cyanide for 2 h, followed by dehydration in an ascending graded series of ethanol and acetone, i.e. 25%, 50%, 75%, 95% and 100% for 10 min at each concentration. Cells were then infiltrated with resins by passing them through three changes of mixture, comprised of a combination of acetone, ethanol and araldite. The following day, cells were infiltrated with four changes of absolute embedding media (araldite) with 1 h incubation at room temperature, 40°C, 45°C and 50°C. Following the last spin, the cell pellet was resuspended in 100–200 µl of araldite. The mixture was embedded using the BEEN capsule (size 3) and was incubated at 60°C for 24 h to allow polymerization. Samples were trimmed with an ultramicrotome (Reichert-Jung, New York, USA) and the sections were stained with 2% uranyl acetate and fixed with lead citrate. The stained sections were viewed under the transmission electron microscope Philip EM 208 and images were captured digitally with a dual view digital camera (Gatan, Werrendale, USA).

**Figure 3:**
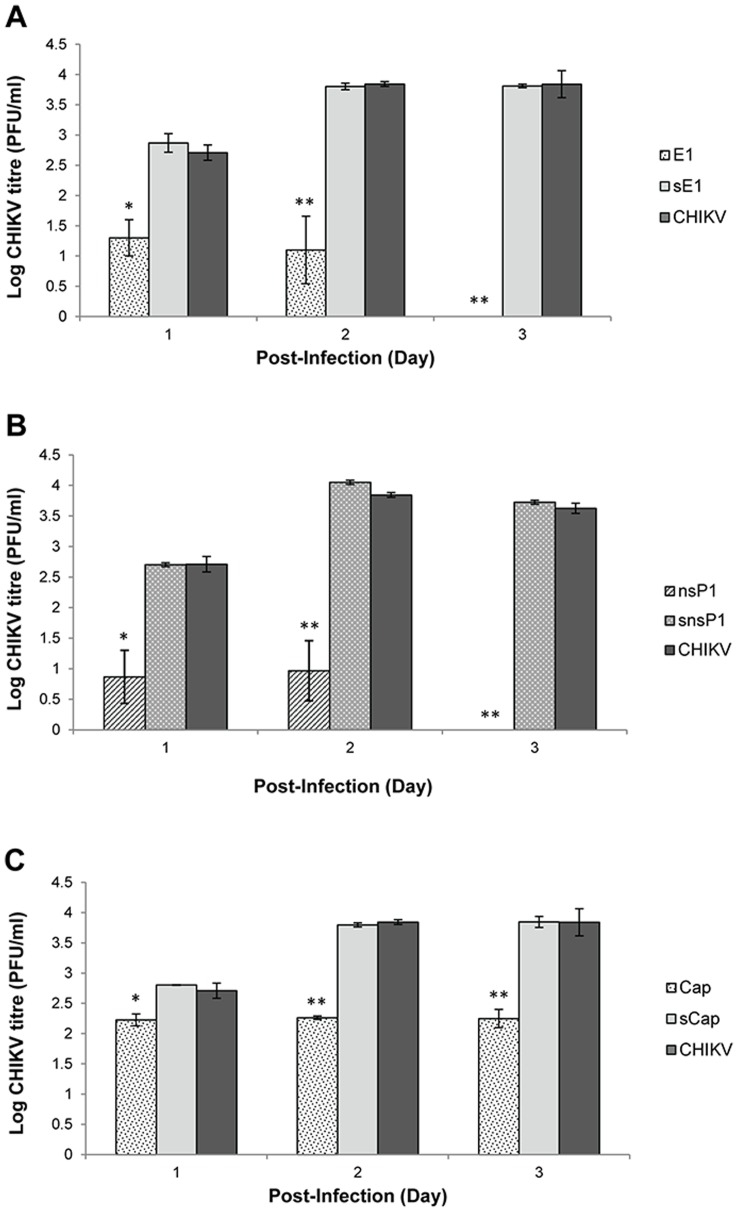
Quantification of CHIKV titre produced from shRNA-expressing HeLa cell clones by viral plaque assays. (**A**) There was an increasing trend of inhibitory effect against CHIKV replication in stable shRNA E1 cell clones relative to the non-transfected HeLa control at Day 1, 2 and 3 p.i. (**B**) Similar anti-CHIKV inhibitory trend was observed in stable shRNA nsP1 cell clones relative to the non-transfected HeLa control. Notably, both shRNA E1 and nsP1 induced complete inhibition of CHIKV infectious viral titre at Day 3 p.i. (**C**) There was a lower inhibitory trend against CHIKV replication in stable shRNA Capsid cell clones at Day 1–3 p.i. relative to the non-transfected cells. Compared to shRNA E1 and nsP1, shRNA Capsid did not produce a complete inhibition against CHIKV replication at Day 3 p.i. The average ±S.E. (standard error) is expressed from three independent set of experiments. Using Student’s T-test analysis, *indicates significant difference (p<0.05) and **indicates a greater significant difference (p<0.005) from control set.

### SINV and DENV Infection on Stable shRNA Cell Clones

shRNA E1, scrambled shRNA E1 (sE1) and non-transfected HeLa cells were seeded at 80–90% confluency on 24-well plates. On the following day, cells were infected with SINV and DENV at M.O.I. 1, respectively. SINV-infected cells were incubated at 37°C in 5% CO_2_ for 1.5 h, with gentle rocking at every 15 min while DENV-infected cells were subjected to same treatment except for longer incubation of 2 h. Residual unbound viruses in the virus-infected wells were washed off twice with PBS and cells were incubated with DMEM media with 2% FCS at 37°C in 5% CO_2_. Supernatants were then harvested at Day 1, 2 and 3 p.i. for viral plaque assays.

### Screening of CHIKV Resistant Mutants to shRNA-E1 Inhibitory Effect

Stable HeLa cell clones expressing shRNA E1 were seeded at 90% confluency in T25 cm^2^ flasks and infected with CHIKV at M.O.I. 1. Supernatants were harvested at Day 2 p.i. when CPE was observed. 500 µl of the supernatant was then inoculated to freshly seeded shRNA E1 cells for next passage of CHIKV culture. This serial passage of CHIKV was continued for 50 rounds. The supernatants from the 15^th^ and 50^th^ passages were then subjected to plaque purification assay to quantitate the virus titre of any potential shRNA-E1 resistant CHIKV mutants. Briefly, BHK cells were seeded in 6-well plates at 90% confluency and cells were infected with the 15^th^ or 50^th^ CHIKV passage supernatant, respectively. 5% agarose overlay was added after infection and cells were monitored for plaque formation. Visible plaques were isolated by sterile micropipettes, resuspended in fresh media and vortexed for 10 min to ensure maximum release of virus progeny from the agarose. The re-supended supernatants were then inoculated on confluent BHK cells in T25 cm^2^ flasks for another round of virus amplication. When extensive CPE was observed, CHIKV supernatants were harvested. From the supernatants, CHIKV RNA was extracted, purified and sequenced for the shRNA E1 target gene.

**Figure 4:**
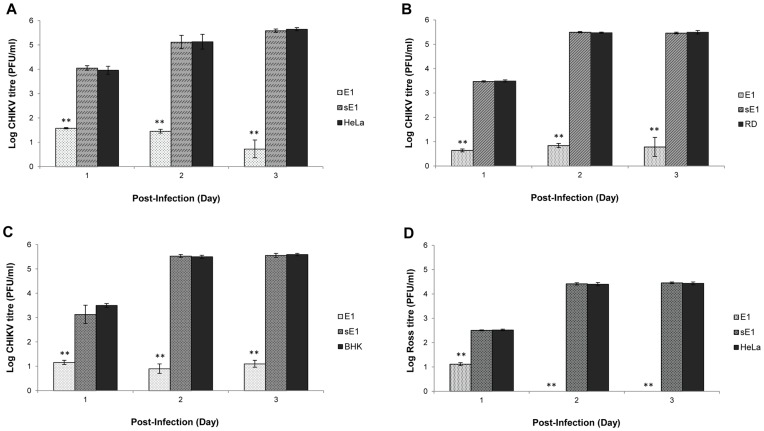
Quantification of CHIKV titre produced from shRNA E1-expressing cell clones. (**A**) At higher M.O.I. 10 of CHIKV infection, shRNA E1 expression showed a strong suppression against CHIKV replication in shRNA HeLa cell clones as compared to the non-transfected and sE1-expressing cell clones. (**B**) **&** (**C**) Similar inhibitory trend of low CHIKV production was noted at Day 1–3 p.i. in RD and BHK cell clones with shRNA E1 activity. (**D**) Broad-spectrum silencing effect of shRNA E1 was notably significant at Day 2 and 3 p.i. where there was complete inhibition on CHIKV Ross strain of the ECSA genotype. The average ±S.E. (standard error) is expressed from three independent set of experiments. Using Student’s T-test analysis, *indicates significant difference (p<0.05) and **indicates a greater significant difference (p<0.005) from control set.

### Virus Protection Assay in CHIKV Murine Model

Suckling C57BL/6 mice of 6 days old (*n = 5*) were inoculated intraperitoneally (i.p.) with three doses of plasmid-shRNA E1, namely, 10, 30 and 60 µg, respectively. At 24 h following treatment with shRNA, mice were subjected to CHIKV infection by i.p. inoculation with 50 µl of CHIKV Singapore/07/2008 strain (containing 10^6^ PFU/ml of CHIKV in RPMI medium supplemented with 2% FCS). Control treatments using 50 µl of scrambled shRNA E1 (sE1) and mock-infection using 50 µl of sterile PBS were also i.p. inoculated into suckling C57BL/6 mice. For the CHIKV-infected groups, mice were monitored daily for signs of flaccid paralysis and mortality for up to 15 days p.i. Any lethality was recorded. All procedures in handling of mice were carried in accordance to the approved protocol from the National University of Singapore Institutional Animal Care and Use Committee (Protocol No. 023/12).

## Results

### Sequence Homology and RNA Structural Analysis of shRNA Target Sites

Nucleotide BLAST alignment of the selected shRNA Capsid, E1 and nsP1 gene target sequences (21-mer) to the complete genomic sequence of the different geographical isolates of CHIKV revealed a 100% homology in gene composition (Table 2). Scrambled sequences of shRNA Capsid, E1 and nsP1 were also verified and were noted to share no considerable sequence identity to the genome of these CHIKV isolates (data not shown). Additionally, shRNA Capsid, E1 and nsP1 were predicted to exhibit minimal complex secondary RNA structural folding (Figure 1). These bioinformatic approaches may assist to ensure that the design of the anti-CHIKV shRNAs to have the potential to induce a broad-spectrum and effective gene silencing activity against different geographical CHIKV strains.

**Figure 5:**
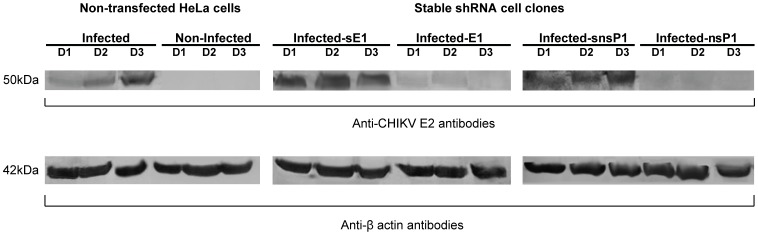
Detection of CHIKV E2 protein expression from HeLa cell clone lysates. Western blotting revealed considerable knockdown of viral E2 protein expression in CHIKV-infected stable shRNA E1 and nsP1 cell clones relative to CHIKV-infected shRNA scrambled E1 (sE1) and shRNA scrmabled nsP1 (snsP1) cell clones and non-transfected HeLa cells at Day 1, 2 and 3 (D1, D2, D3) p.i. β-actin was used as a loading control to ensure equal protein loading in all wells.

### Establishment of CHIKV Growth Kinetics in HeLa Cell Line

In order to determine a suitable time point for investigating the anti-CHIKV efficacy of shRNA expressed in the stable cell clones, CHIKV growth kinetic in non-transfected HeLa cells was established at the start of the study. CHIKV replication in HeLa S3 cell line was quantitated by performing viral plaque assays using viral supernatants harvested at fixed time points of 0 h, 6 h, 12 h, 24 h, 48 h and 72 h p.i. As shown in Figure 2, CHIKV infection in HeLa cells at M.O.I. 1 have resulted in a rapid production of infectious virions from 12 h to 48 h p.i. The peak titre of 10^3.8^ PFU/ml was reached on 48 h p.i. and was sustained to 72 h p.i. Overall, this indicates that HeLa cells are permissive to CHIKV infection and replication. This finding was in agreement with a previous work which demonstrated the susceptibility of HeLa cells to CHIKV infection [21]. Contrary to the notion that alphaviruses exhibit a rapid and efficient propagation within 24 h p.i. *in vitro* [16], [21], we found that the highest infectious CHIKV titre was reached and sustained between 48 h and 72 h p.i. in HeLa cells. CHIKV growth kinetic data with other vertebrate cell lines such as BHK and Vero cells also showed late replication characteristics (data not shown). Thus in this study, Day 1, 2 and 3 p.i. time points were selected to assess the antiviral efficacy of shRNAs in the stable shRNA cell clones and HeLa cell controls.

**Figure 6:**
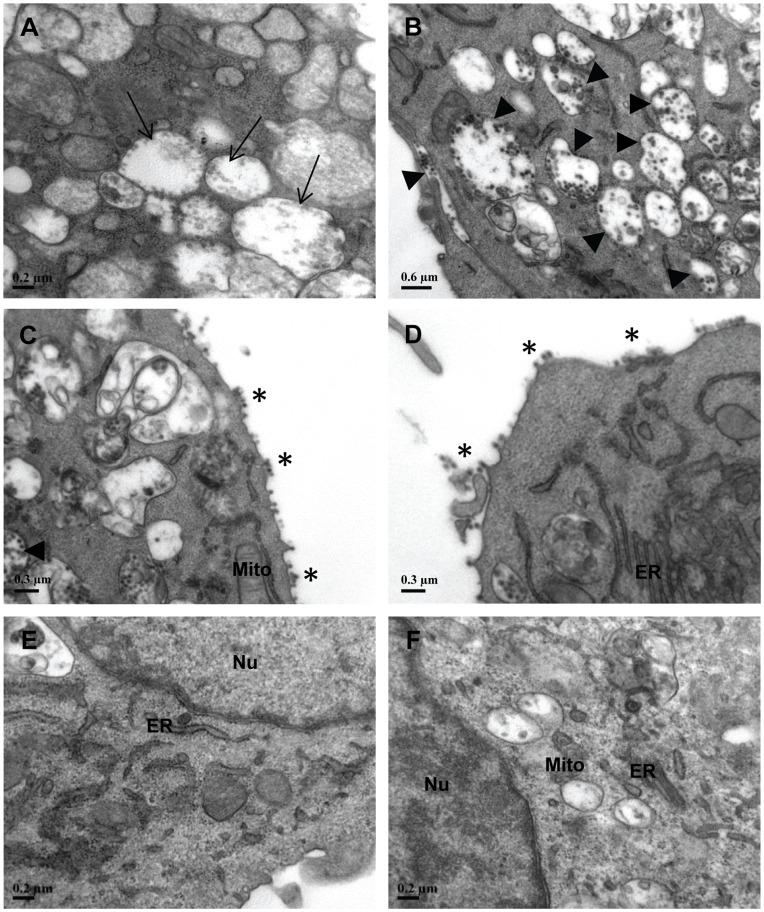
Ultrastructural analysis of shRNA cell clones and non-transfected HeLa cells upon CHIKV infection. At Day 3 p.i., extensive CHIKV replication in non shRNA-expressing HeLa cells was detected with (**A**) formation of viral replication complexes (Cytopathic vacuoles type I, CPV-I →) as well as (**B**) numerous CPV-II (▸) containing CHIKV particles in the cytosol. Similar trend of CHIKV infection was observed in (**C**) stable shRNA scrambled E1 and (**D**) shRNA scrambled nsP1 cell clones where CHIKV virions were detected to be budding off (*) at the plasma membrane. (**E**) Stable shRNA E1 and (**F**) nsP1 cell clones maintained healthy morphology in their membranous organelles structure. There was an absence of CHIKV-induced replication complexes and virus particles in these cell clones. ER, endoplasmic reticulum; Mito, mitochondrion; Nu, nuclei.

### Decreased CHIKV Titre in shRNA-expressing Cell Clones

To evaluate the antiviral capabilities of shRNA Capsid, E1 and nsP1, viral plaque assay was carried out to quantitate the amount of infectious virus progeny produced upon CHIKV infection in shRNA-expressing HeLa cell clones and non-transfected HeLa cells. Following CHIKV infection of these cell clones at M.O.I. 1, viral supernatants were harvested at Day 1, 2 and 3 p.i. and subjected to viral plaque assay. Compared to the CHIKV infected-HeLa cell control, stable shRNA E1 cell clones produced a significant inhibitory trend against CHIKV replication from Day 1–3 p.i (Figure 3A). Infectious CHIKV titre in shRNA E1 cell clones demonstrated a significant reduction (p<0.05) of 1.5 log PFU/ml at Day 1 p.i., and a further significant decrease of 2.8 log PFU/ml at Day 2 p.i. when compared to the infected-HeLa cells without shRNA treatment. Notably, there was a complete inhibition against CHIKV replication at Day 3 p.i. Similarly, shRNA nsP1 showed a sustained anti-CHIKV inhibitory effect from Day 1–3 p.i. CHIKV replication was strongly suppressed, with low infectious titre of less than 1 log PFU/ml being observed at Day 1 and 2 p.i. (Figure 3B). Remarkably, shRNA nsP1 expression in the stable cell clone was also found to exert a significant (p<0.005) and complete inhibition of CHIKV replication at Day 3 p.i, compared to the non-transfected cells. Relative to shRNA nsP1 and E1, shRNA Capsid exhibited a lower anti-CHIKV activity with a sustained production of CHIKV infectious titre at an average of 2.3 log PFU/ml from Day 1–3 p.i. (Figure 3C). This represents a modest level of inhibition by 1.6 log PFU/ml at Day 3 p.i. when compared to the non-transfected cells. Overall, scrambled shRNA Capsid, sE1 and snsP1 cell clones did not produce any significant inhibitory effect against CHIKV replication.

**Figure 7:**
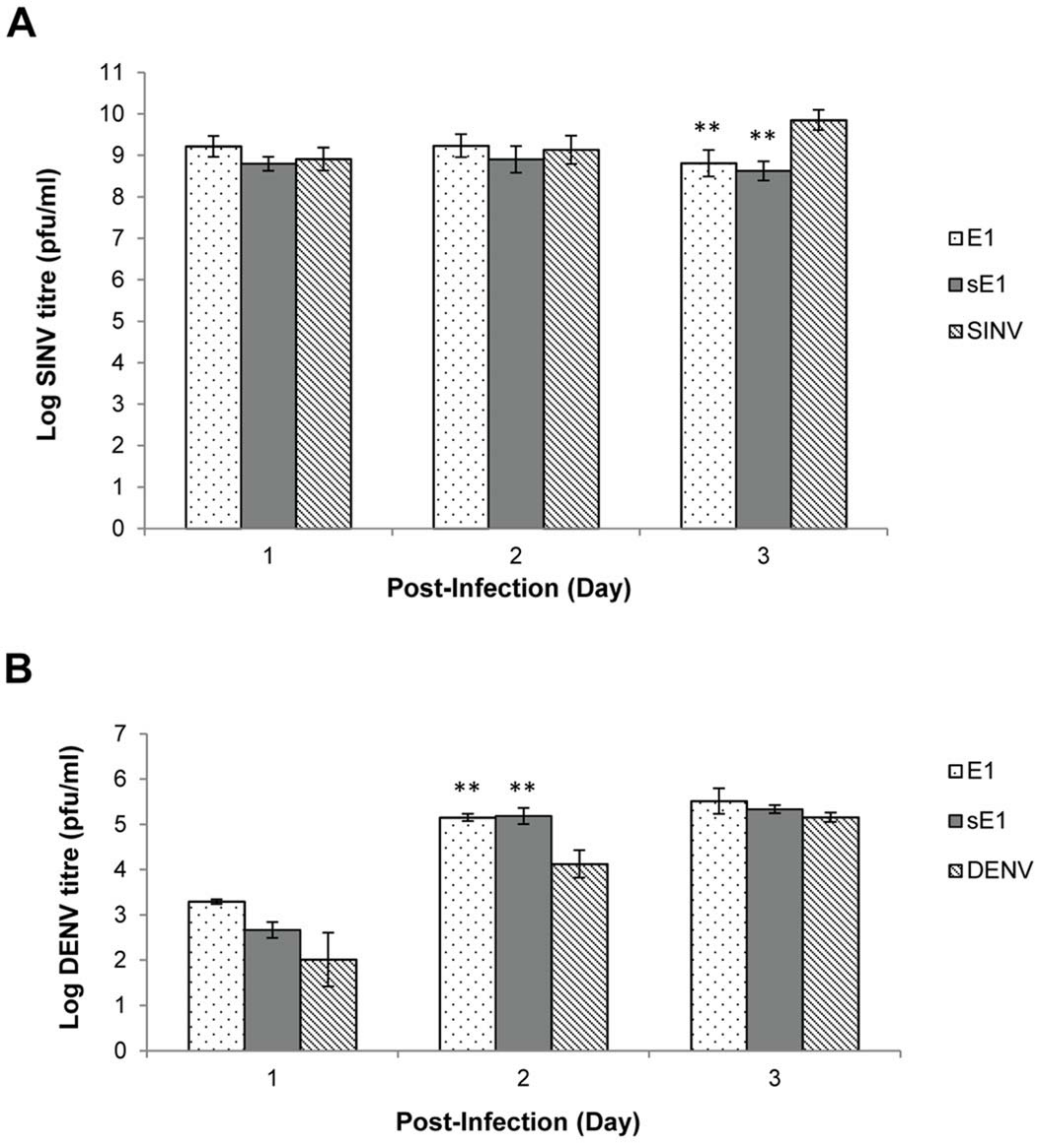
Quantification of SINV and DENV infectious titres produced from shRNA cell clones and non-transfected HeLa cells. (**A**) SINV infection produced consistently high virus yield throughout Day 1–3 p.i., relative to their shRNA scrambled E1 and non-transfected HeLa cell controls. (**B**) DENV infection showed an increasing trend of virus replication during Day 1–3 p.i, relative to their controls. Taken together, both data indicate the non-target specificity of shRNA E1 against SINV and DENV replication. The average ±S.E. is expressed from three independent set of experiments. Using Student’s T-test analysis, *indicates significant difference (p<0.05) and **indicates a greater significant difference (p<0.005) from control set.

As both shRNA E1 and nsP1 showed strong inhibitory effect against the production of CHIKV in stable HeLa cell clones, shRNA E1 was then selected as a representative shRNA to further investigate its anti-CHIKV efficacy. To investigate if the silencing potency of shRNA can be maintained at higher M.O.I. of CHIKV infection, CHIKV infection of stable shRNA cell clones was performed at M.O.I. 10. Relative to the non-transfected HeLa cell controls, there was a strong and sustained reduction of CHIKV infectious titre at Day 1–3 p.i. in shRNA E1 cell clones. Notably, less than 1 log PFU/ml of CHIKV infectious titre was achieved at Day 3 p.i. (Figure 4A). Taking this finding with that at M.O.I. 1, a similar trend of inhibition against CHIKV replication was found to be mediated by shRNA E1 at M.O.I. 1 (Figure 3A) and a high M.O.I. of 10.

**Figure 8:**
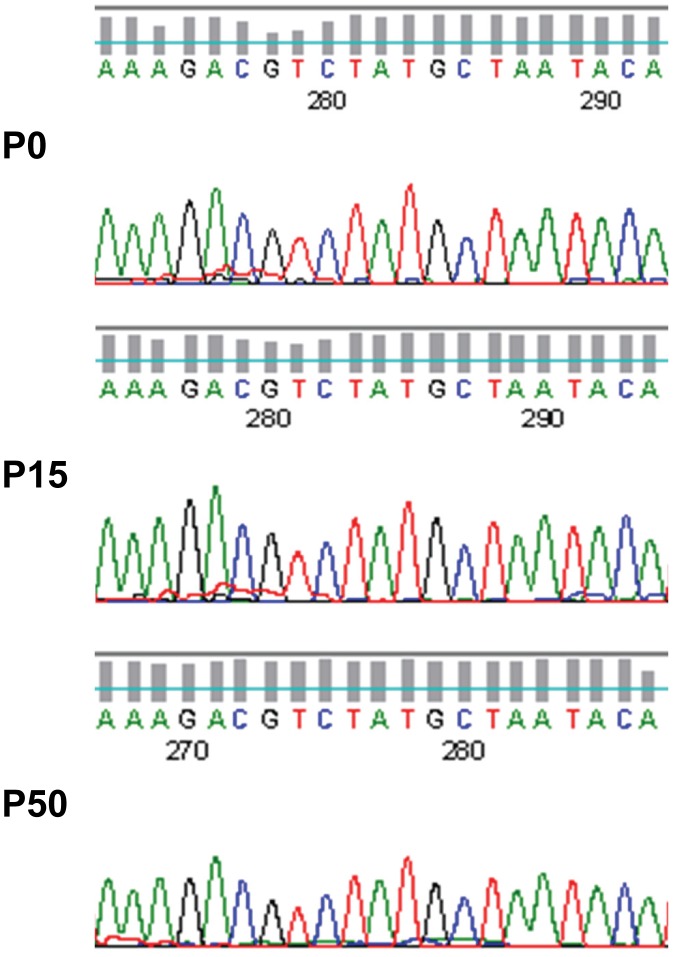
Sequencing of shRNA E1 target region upon extensive passaging of CHIKV in shRNA-expressing cell clones. Continuous maintenance of CHIKV in shRNA E1 cell clones at the 15^th^ (P15) and 50^th^ (P50) passage did not indicate presence of CHIKV resistant mutations in the shRNA target sequence of the viral RNA. Sequences are comparable to the sequence of the initial passage (P0) CHIKV RNA genome.

In order to investigate the anti-CHIKV effect of shRNA in other CHIKV-permissive cell lines, stable RD and BHK cell clones expressing shRNA E1 were established and infected with CHIKV at M.O.I. of 1. shRNA E1 exerted a significant inhibition (p<0.005) against CHIKV replication in both RD and BHK cell clones at Day 1 to 3 p.i. In particular, there was a strong suppression of CHIKV replication by an average of 4.7 log PFU/ml at Day 2 and 3 p.i. as compared to the non-transfected RD cells (Figure 4B). Similarly, shRNA E1 favorably reduced infectious CHIKV titre by an average of 4.5 log PFU/ml at Day 2 and Day 3 p.i. in stable BHK cell clones relative to non-transfected BHK cell controls (Figure 4C). Overall, there was a sustained inhibition against CHIKV replication at Day 2 and 3 p.i in both RD and BHK stable cell clones expressing shRNA E1. This suggests that shRNA-mediated silencing was protective against CHIKV replication in other permissive cell lines (RD and BHK cells) for CHIKV infection.

Furthermore, shRNA E1, nsP1 and Capsid were designed with the aim of inducing broad-spectrum silencing against different geographical CHIKV isolates, including the ECSA, CEA and Asian genotypes. The broad-spectrum activity of shRNA E1 was validated by infection with CHIKV Ross strain of the ECSA genotype. shRNA E1 cell clones showed strong and complete suppression on the replication of CHIKV Ross strain at Day 2 and 3 p.i., relative to the non-transfected HeLa cell controls (Figure 4D).

**Figure 9:**
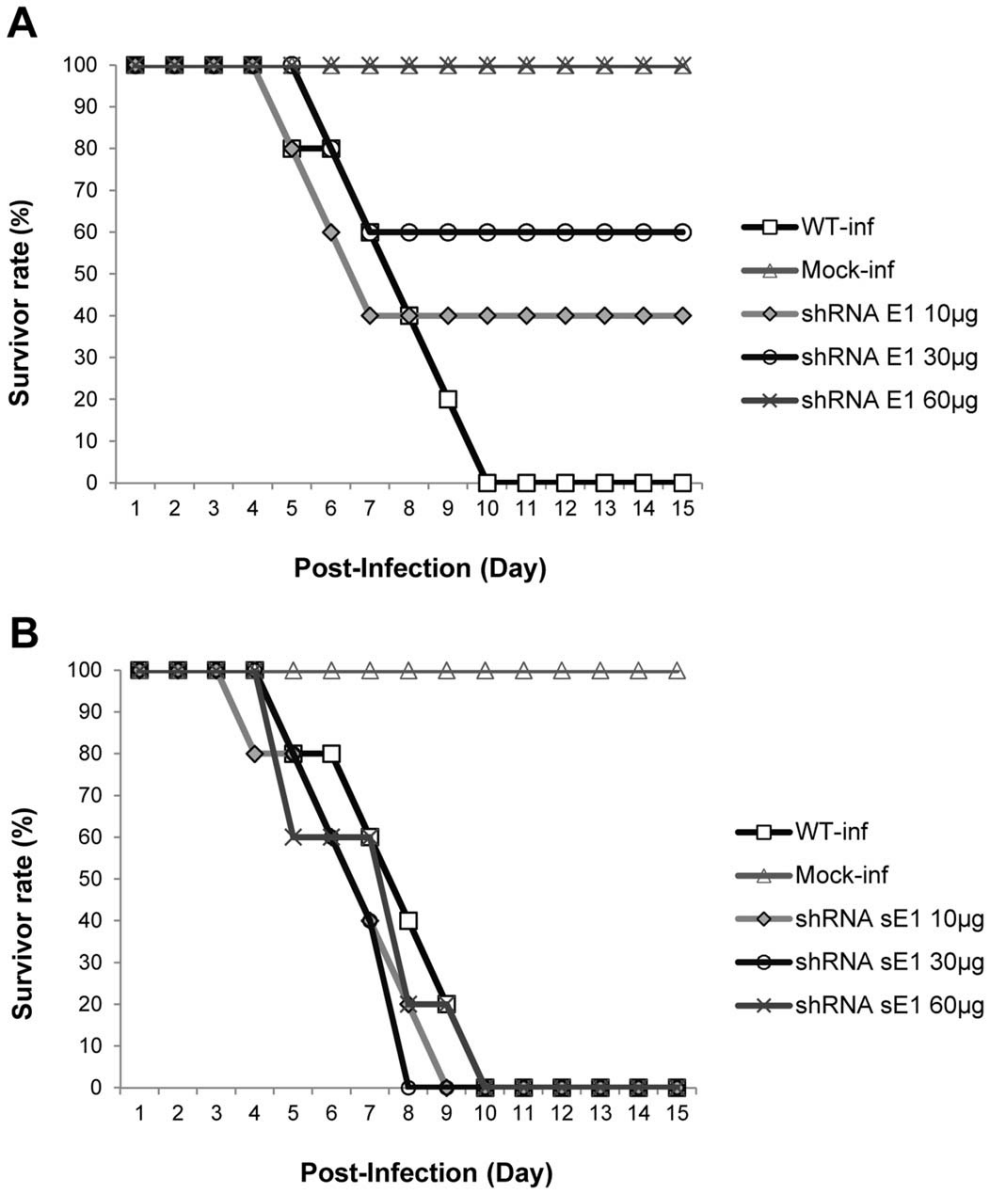
CHIKV infection in mice pre-treated with shRNA E1. In contrast to the wildtype and scrambled E1 (sE1)-treated groups, pre-treatment of mice with single doses of plasmid-shRNA E1 via intraperitoneal (i.p.) route was shown to confer strong protective effect against CHIKV disease (*n = 5* per treatment group). Survival of these shRNA E1-treated mice suggested a dose-dependent inhibition against CHIKV pathology throughout 15 days p.i. CHIKV infection was carried out using 10^6^ PFU. WT refers to the non-treated group; For the mock-inf, sterile PBS was inoculated in replacement of infectious CHIKV; shRNA sE1 refers to the shRNA scrambled E1 plasmid.

### Decreased CHIKV Protein Production in shRNA-expressing Cell Clones

Western blot analysis of CHIKV E2 protein expression levels in CHIKV infected-shRNA HeLa cell clones was performed to further validate the efficacy of shRNA E1 and nsP1 in silencing CHIKV replication and viral protein expression. Following CHIKV infection at M.O.I. 1, shRNA E1 and nsP1 cell clones showed minimal expression of CHIKV E2 protein at Day 1, 2 to 3 p.i., as compared to their scrambled shRNA controls and non-transfected HeLa cells (Figure 5). Notably at Day 3 p.i., shRNA E1 cell clones showed complete suppression of CHIKV E2 protein expression. Similarly, shRNA nsP1 displayed a similar inhibitory trend in CHIKV E2 production when compared to the non-transfected HeLa cells and scrambled nsP1 cell clones, respectively.

### Absence of CHIKV Replication in Ultrastructural Analysis of shRNA-expressing Cell Clones

The above-mentioned findings have consistently showed that CHIKV replication in shRNA-expressing E1 and nsP1 cells were suppressed at both levels of virus progeny yield and CHIKV protein expression. To further examine shRNA activity against CHIKV replication, the ultrastructures of CHIKV-infected cell clones and non-transfected HeLa cells at Day 3 p.i. were analyzed using transmission electron microscopy (TEM). In the viral-infected HeLa and scrambled shRNA cell controls, there was formation of numerous CHIKV replication complexes, namely, the cytopathic vacuoles type I (CPV-I) and type II (CPV-II) in the cytosol (Figure 6A–6C). These viral-induced structures represent sites for the rapid synthesis of CHIKV RNA and protein, thus indicating the presence of extensive CHIKV replication. In the late phase of viral replication, the assembly and budding of mature CHIKV particles from the cell surface membrane of HeLa and scrambled shRNA cell controls were also observed (Figure 6C and Figure 6D). Relative to scrambled shRNAs and HeLa cell controls, there was indeed an absence of CHIKV particles and CHIKV-induced CPE in shRNA E1 and nsP1 cells (Figure 6E and 6F). The intact morphology of cellular organelles was comparable to that of the uninfected HeLa cell control (data not shown). These TEM images strongly support the efficacy of shRNA in the knockdown of CHIKV RNA genome expression and thus the protection in cellular morphology against CHIKV replication.

### Anti-CHIKV shRNA has no Antiviral Activity against the Replication of Sindbis and Dengue Viruses

To evaluate if the shRNA E1 construct was targeting CHIKV replication specifically, stable shRNA E1 cell clones were infected with either a closely related alphavirus, Sindbis virus (SINV) or a flavivirus, Dengue virus (DENV) at M.O.I. 1, respectively. Stable shRNA E1 cell clones were used as a representative shRNA-expressing cell clone for this experiment. Plaque assay results revealed that anti-CHIKV shRNA E1 did not exert any considerable inhibition against SINV replication in stable cell clones when compared against the non-transfected HeLa controls at Day 1 and 2 p.i. (Figure 7A). However, at Day 3 p.i., both SINV infected-shRNA E1 and sE1 cell clones demonstrated a slight reduction of 1 log PFU/ml in infectious SINV titre production when compared to the infected-HeLa cells. Nevertheless, given that complete inhibition of CHIKV replication was observed in CHIKV infected-shRNA E1 and nsP1 cell clones (Figure 3A and 3B), the 1 log PFU/ml decrease in SINV infectious titre was considered to be minimal. It is unlikely that the inhibition of SINV replication was due to a shRNA-specific effect against SINV replication. A similar non-inhibitory trend of shRNA E1 was also observed in DENV-infected shRNA E1 cell clones (Figure 7B). Taken together, these data suggest the lack of specificity between the anti-CHIKV shRNA E1 target sequence and the SINV and DENV genomic sequences. Hence, shRNA E1 was shown to exert a high target-specificity in mediating specific knockdown of CHIKV replication.

### Mutagenic Study of CHIKV Resistance against shRNA E1 Target Sequence

As CHIKV has a high capability to evolve to a higher mutagenic forms by accumulating genomic mutations [22], [23], the long-term efficacy of anti-CHIKV shRNAs in targeting CHIKV replication was evaluated. CHIKV was maintained in 50 continuous passages in stable shRNA E1 cell lines. Supernatants harvested were plaque-purified, sequenced and analyzed for the occurrence of any mutation in the shRNA target site. Here, we found out that the shRNA E1 target sequence of CHIKV was 100% conserved after 15 and 50 rounds of CHIKV passage in stable shRNA E1 cell clones (Figure 8). Therefore, there is a low possibility of CHIKV-resistant mutants to arise from this shRNA-based antiviral strategy.

### Pre-treatment with shRNA E1 Protected Mice against CHIKV Infection

To substantiate our *in vitro* findings on the anti-CHIKV efficacy of shRNA, we further evaluated the activity of shRNA E1 in a murine model using the CHIKV Singapore/07/2008 strain. Minimal cytotoxicity of the different dosages of shRNA E1 and scrambled shRNA sE1 upon inoculation into the suckling mice was confirmed by measuring the level of lactate dehydrogenase in the sera of suckling mice (data not shown). Suckling mice were pre-treated with plasmid-shRNA E1 or plasmid-shRNA scrambled sE1 at single doses of 10, 30 and 60 µg, respectively, followed by i.p. inoculation with 10^6^ PFU of CHIKV at 24 h post-treatment. Mice pre-treated with shRNA E1 were found to develop resistance against CHIKV disease onset relative to non-treated and the scrambled shRNA E1 controls. Of note, pre-treatment with shRNA E1 at 30 µg produced 60% survival after Day 7 p.i., while a higher dose of 60 µg conferred and sustained 100% survival of the mice for 15 days p.i (Figure 9A). In contrast, non-treated mice and the scrambled shRNA sE1-treated mice showed severe mortality of more than 40% at Day 7 p.i. (Figure 9B), with symptoms of flaccid paralysis including difficulty walking and dragging of hind limbs. Complete mortality of these control mice was observed by Day 8–10 p.i.

## Discussion

In this study, a novel approach using plasmid-based shRNA expression to target CHIKV Capsid, E1 and nsP1 genes was investigated for its potential to silence CHIKV replication in stable shRNA-expressing cell clones. Antiviral studies on mammalian cells using customized siRNAs have reported inhibitory efficacy of these siRNAs against CHIKV replication [16] and the replication of other alphaviruses such as Venezuelan Equine Encephalitis Virus (VEEV) and Semliki Forest Virus (SFV) [17], [18]. In particular, siRNAs targeting CHIKV E1 and nsP3 were effective in suppression of *in vitro* CHIKV replication [16]. This highlights the potential application of RNA interference technology as a future antiviral for CHIKV. However, effective suppression of CHIKV replication could not be sustained by siRNAs on a long term basis due to the rapidly replicating nature of alphaviruses in infected cells [16]. Moreover, siRNAs are highly susceptible to intracellular degradation [24], allowing only a transient knockdown of viral mRNA expression. In view of these limitations, this study utilized a constitutive siRNA production system where shRNAs were expressed from a stable DNA plasmid under the control of CMV promoter. Following transfection of the plasmid-shRNA into HeLa cells, stable cell clones were selected and infected with CHIKV. Following CHIKV infection, the anti-CHIKV efficacies of these shRNAs were evaluated at Day 1, 2 and 3 p.i. when peak CHIKV production was observed to occur.

Data obtained from viral plaque assay, Western blotting and TEM, collectively demonstrated that shRNA E1 and nsP1 exhibited sustained inhibitory activity against the replication of CHIKV upon infection at M.O.I. 1 in stable HeLa cell clones at Day 1 and Day 2 p.i. Notably, shRNA E1 and nsP1 have individually produced a strong inhibition against CHIKV replication at Day 3 p.i (Figure 3A). At this time point, both shRNA E1 and nsP1 remarkably demonstrated 100% inhibition of infectious CHIKV production. In addition, shRNA E1 was shown to suppress CHIKV replication even at higher M.O.I. of 10 (Figure 4A). Moreover, at the viral protein expression level, both shRNA E1 and nsP1 resulted in a considerable reduction of CHIKV E2 protein expression (Figure 5). Contrary to the absence of infectious virions as determined by plaque assay, there was some viral E2 protein detected in both E1 and nsP1 stable cell clones at Day 3 p.i. This could have been due to residual CHIKV E2 proteins undergoing gradual degradation in the shRNA cell clones at Day 3 p.i. Nevertheless, the complete silencing of CHIKV replication was further supported by observations at the ultrastructural level of these stable cell clones (Figure 6). There was no sign of CHIKV replication in the stable shRNA E1 and nsP1 cell clones at Day 3 p.i., in contrast to the extensive CPE (data not shown) and formation of numerous CHIKV-induced replication complexes CPV-I and II on the membraneous organelles in non-transfected HeLa cells (Figure 6A and 6B) and scrambled shRNA cell clones (Figure 6C). Being a unique morphological characteristic to the *Togaviridae* family, CPVs are modified endosomal and lysosomal structures that represents the sites of viral RNA replication and possibly RNA translation and nucleocapsid assembly [25]. Hence, the lack of CPV-II formation in the late phase of virus replication (Day 3 p.i.) provided strong evidence of shRNA-mediated inhibition against CHIKV replication. Our findings were also consistent with similar studies that have demonstrated strong antiviral activity of plasmid-based shRNA against West Nile virus [26], Influenza A virus [27], Hepatitis B virus [28] and SFV [18].

Notably, one of the critical factors for an effective shRNA-mediated silencing of CHIKV replication is the design of an effective shRNA construct. In this study, optimal screening and selection of a promising target site in the coding sequence of E1 and nsP1 CHIKV genes was carried out. Any potential secondary structure formation of the shRNA E1 and nsP1 products and CHIKV target gene sequences was predicted by mfold webserver [29] (hosted at http://mfold.rna.albany.edu/?q=mfold/RNA-Folding-Form). It was observed that shRNA E1 and nsP1 target sites in the CHIKV RNA genome and all anti-CHIKV shRNAs did not exhibit complex structural folding (Figure 1). This ensured the accessibility of the siRNA/RISC complex to the respective target sequences to mediate viral RNA cleavage. In contrast, Capsid target sequence in the CHIKV genome was predicted to form a considerable secondary structure, which may have lowered its accessibility and binding to Capsid siRNA, leading to the modest inhibitory effect observed for shRNA Capsid relative to shRNA E1 and nsP1 against CHIKV replication. In addition, the shRNA was constructed based on its target site in having 100% homology to several geographical isolates of CHIKV. This conferred an advantage of a broad-spectrum silencing efficacy of the anti-CHIKV shRNA constructs.

The broad-spectrum design incorporated into the design of plasmid-shRNA E1 was verified in stable shRNA-expressing RD and BHK cell clones. As muscle cells and fibroblasts are well-established as targets for CHIKV infection [21], [30], extensive CHIKV replication was noted in scrambled shRNA E1 cell clones, non-transfected RD cells and BHK cells at Day 1–3 p.i (Figure 4B and 4C). In contrast, shRNA E1 was found to significantly suppress CHIKV replication (Figure 4B and 4C). This indicates that anti-CHIKV efficacy of shRNA was non-cell type specific. In addition, stable shRNA E1 cell clones exhibited significant inhibition against CHIKV Ross replication (Figure 4D). This further highlights the anti-CHIKV effect of shRNA against CHIKV strain of the ECSA genotype.

As plasmid-shRNA expression [31] and CHIKV infection are known to induce cellular interferon (IFN) response [32], [33], it is plausible that the decreased virus production was not specifically due to the shRNA silencing activity on CHIKV RNA. However, studies have shown that HeLa cells lack Type I IFN expression which is present in other mammalian cells such as the macrophages and lymphocytes [34], [35], [36]. HeLa cells are found to constitutively express IFN-ε, an isoform which has not been shown to be directly stimulated upon virus challenge. Moreover, other IFN isoforms such as IFN-α and IFN-β, which are typically up-regulated during virus infection, were minimally detected in infected HeLa cells [34], [36]. Synthetic siRNA were also not shown to induce significant antiviral response in siRNA-transfected HeLa cells [37]. Thus, the considerably low CHIKV titre in infected cell clones in this study could largely be attributed to effective shRNA-mediated silencing of CHIKV replication.

The stringency of siRNA-target base pairing may impose a selective pressure on CHIKV to form shRNA-resistant mutant strains. These mutant strains may acquire favourable mutations in the shRNA target sequence so as to evade the shRNA inhibitory effect, thus rendering shRNA to be ineffective. This has been demonstrated in siRNA studies on HIV and Hepatitis C virus [38], [39]. However, in this study, CHIKV replication in stable shRNA E1 cell passages did not induce mutation to the specific shRNA target region in the CHIKV genome. There was 100% complete conservation of the CHIKV RNA sequence analyzed (Figure 8), suggesting that no CHIKV resistant strains were generated under long term passages in shRNA-expressing cell clones.

With the promising findings obtained from *in vitro* studies, the antiviral activity of shRNA was further evaluated for its efficacy *in vivo* using the murine model for CHIKV infection. C57BL/6 mice suckling mice were used as they have previously been established as susceptible murine models for CHIKV infection [40]. Complete protection against CHIKV pathology was demonstrated in mice pre-treated with 60 µg of shRNA E1. Survival of these pre-treated mice was observed up to Day 15 p.i. as compared to the manifestation of CHIKV symptoms and CHIKV-induced lethality in non-treated and scrambled shRNA-treated mice. A dose-dependent resistance against CHIKV replication was also conferred by pre-treatment with increasing doses of shRNA E1 as opposed to pre-treatment with the scrambled shRNA sE1. Hence, these data provides strong evidence on the anti-CHIKV efficacy of shRNA *in vivo* and reinforces the potentially usefulness of shRNA in clinical settings of CHIKV infection.

In conclusion, this study has demonstrated that a plasmid-based shRNA expression antiviral technology directed against CHIKV E1 and nsP1 was effective in producing sustained inhibition against CHIKV replication. These findings open further possibilities of plasmid-based shRNA E1 as an effective antiviral strategy against CHIKV infection.

## Supporting Information

Figure S1 **Simplified overview of the RNAi pathway mediated by shRNA to silence CHIKV replication.** In this study, three shRNAs were specifically designed to target against CHIKV Capsid, E1 and nsP1 RNA and these shRNAs were expressed in stable HeLa cell clones. Firstly, shRNA-plasmid construct is introduced into the cell by liposomal transfection (**1**). Upon plasmid expression in the nucleus, small-hairpin RNA (shRNA) is formed (**2**) and is subsequently processed by cytoplasmic Dicer enzyme to siRNAs of 21–23 b.p. (**3, 4**). One of the siRNA strands (antisense strand) is loaded into a RNA-induced silencing complex (RISC) which contains an endonuclease [19]. This results in the formation an activated RNA silencing machinery known as siRNA/RISC (**5**). Following endocytosed entry of CHIKV virion, the single-stranded viral RNA genome is released into the host cytosol (**6**). The siRNA guide strand in the RISC primes the complex to recognize and degrade the target CHIKV RNA (**7**), leading to the overall knockdown of CHIKV protein expression and suppression of viral replication in the infected cell (**8**).
(TIF)

Figure S2 **ClustalW2 alignment of the query pSilencer-shRNA sequence with the designed CHIKV shRNA E1 sequence.** *indicates exact nucleotide match; Sequences 1) underlined are the SV40 polyA signals, 2) highlighted are the bottom strand of shRNA oligonucleotide and 3) in bold are the CMV promoter sequence.
(TIF)

Figure S3 **DNA sequencing analysis of shRNA-plasmid construct.** shRNA E1 oligonucleotide construct (55-mer) cloned into pSilencer vector was validated by DNA sequencing to be in correct sequence composition and orientation. The shRNA target sequence is outlined.
(TIF)
