# Supplementary figures and images for: Expression of Plasmid-Based shRNA against the E1 and nsP1 Genes Effectively Silenced Chikungunya Virus Replication
Source: PLoS One. 2012 Oct 8;7(10):e46396. doi: 10.1371/journal.pone.0046396 (PMC3466284; doi:10.1371/journal.pone.0046396)

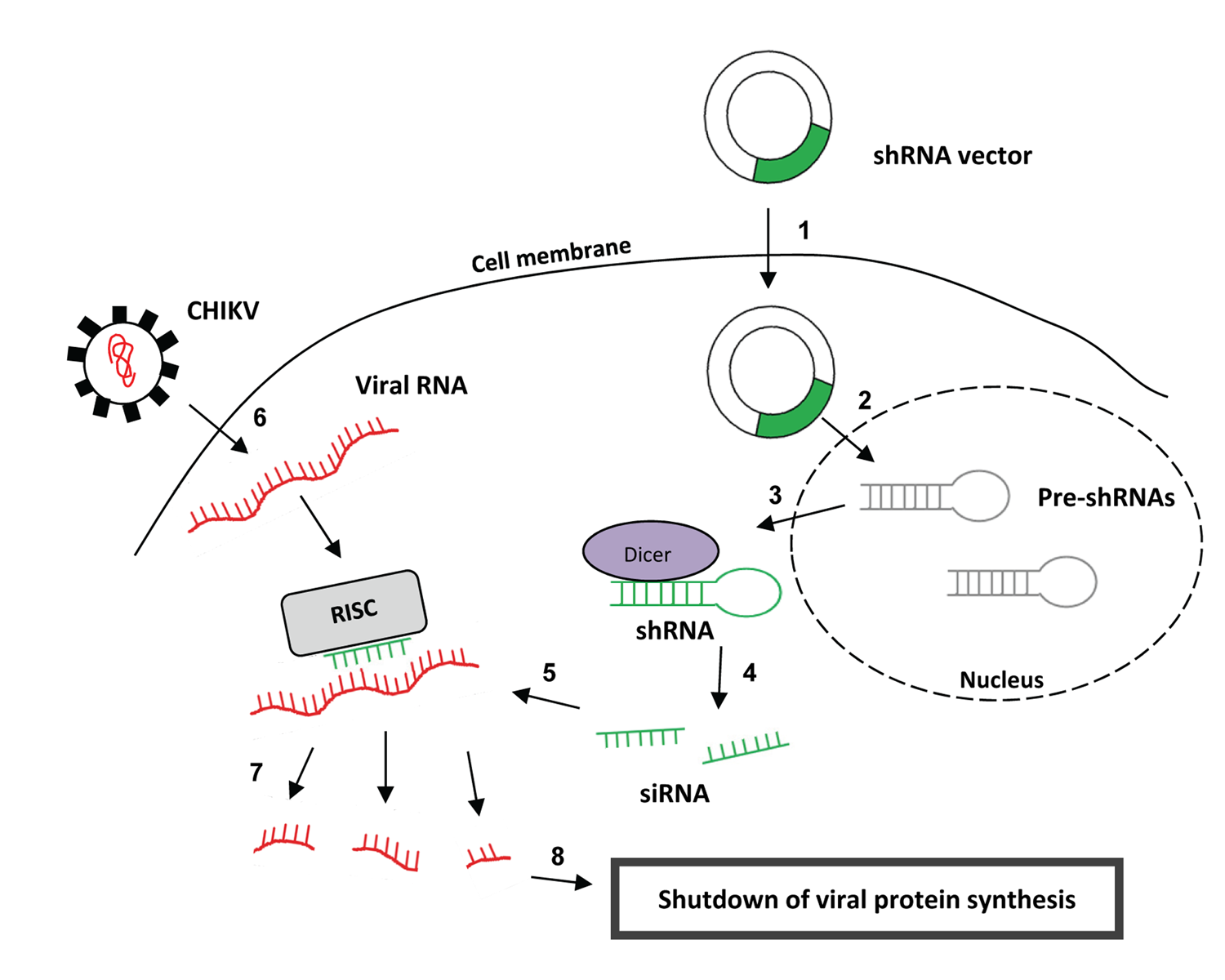

Supplement: Figure S1 — Simplified overview of the RNAi pathway mediated by shRNA to silence CHIKV replication. In this study, three shRNAs were specifically designed to target against CHIKV Capsid, E1 and nsP1 RNA and these shRNAs were expressed in stable HeLa cell clones. Firstly, shRNA-plasmid construct is introduced into the cell by liposomal transfection (1). Upon plasmid expression in the nucleus, small-hairpin RNA (shRNA) is formed (2) and is subsequently processed by cytoplasmic Dicer enzyme to siRNAs of 21–23 b.p. (3, 4). One of the siRNA strands (antisense strand) is loaded into a RNA-induced silencing complex (RISC) which contains an endonuclease [19]. This results in the formation an activated RNA silencing machinery known as siRNA/RISC (5). Following endocytosed entry of CHIKV virion, the single-stranded viral RNA genome is released into the host cytosol (6). The siRNA guide strand in the RISC primes the complex to recognize and degrade the target CHIKV RNA (7), leading to the overall knockdown of CHIKV protein expression and suppression of viral replication in the infected cell (8). (TIF) [file pone.0046396.s001.tif]

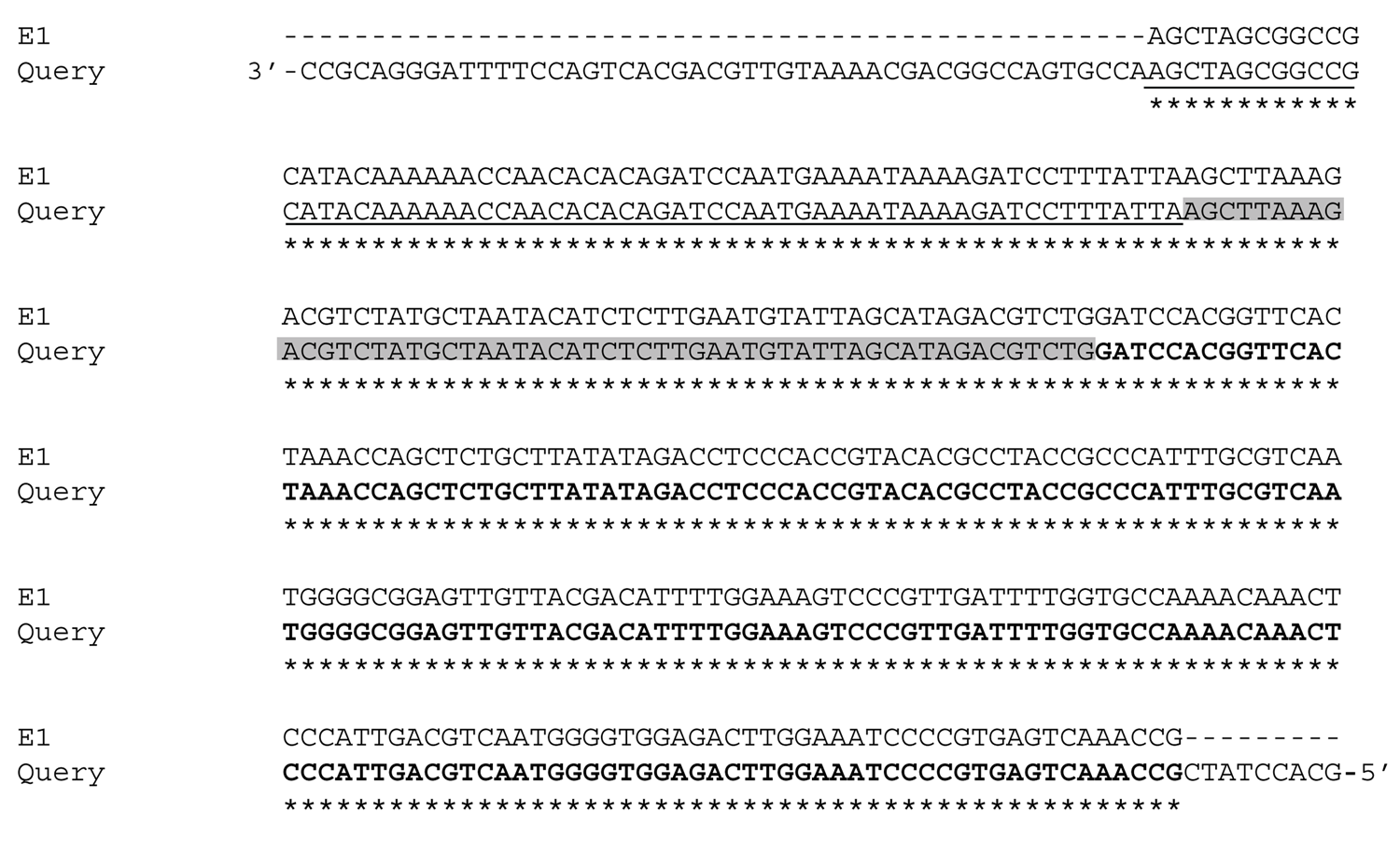

Supplement: Figure S2 — ClustalW2 alignment of the query pSilencer-shRNA sequence with the designed CHIKV shRNA E1 sequence. *indicates exact nucleotide match; Sequences 1) underlined are the SV40 polyA signals, 2) highlighted are the bottom strand of shRNA oligonucleotide and 3) in bold are the CMV promoter sequence. (TIF) [file pone.0046396.s002.tif]

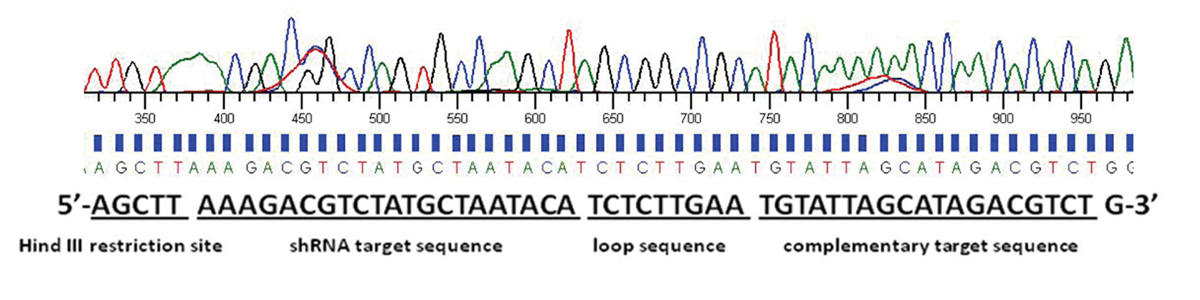

Supplement: Figure S3 — DNA sequencing analysis of shRNA-plasmid construct. shRNA E1 oligonucleotide construct (55-mer) cloned into pSilencer vector was validated by DNA sequencing to be in correct sequence composition and orientation. The shRNA target sequence is outlined. (TIF) [file pone.0046396.s003.tif]
